# Assessing and reducing PET radiotracer infiltration rates: a single center experience in injection quality monitoring methods and quality improvement

**DOI:** 10.1186/s12880-020-0408-3

**Published:** 2020-01-10

**Authors:** Dustin R. Osborne, Shelley N. Acuff, Michael Fang, Melissa D. Weaver, Yitong Fu

**Affiliations:** University of Tennessee Graduate School of Medicine, 1924 Alcoa Highway, Knoxville, TN 37920 USA

**Keywords:** PET, Positron emission tomography, Infiltration, Injection quality, Quality improvement

## Abstract

**Background:**

Successful injection of radiolabeled compounds is critical for positron emission tomography (PET) imaging. A poor quality injection limits the tracer availability in the body and can impact diagnostic results. In this study, we attempt to quantify our infiltration rates, develop an actionable quality improvement plan to reduce potentially compromised injections, and compare injection scoring to PET/CT imaging results.

**Methods:**

A commercially available system that uses external radiation detectors was used to monitor and score injection quality. This system compares the time activity curves of the bolus relative to a control reading in order to provide a score related to the quality of the injection. These injection scores were used to assess infiltration rates at our facility in order to develop and implement a quality improvement plan for our PET imaging center. Injection scores and PET imaging results were reviewed to determine correlations between image-based assessments of infiltration, such as liver SUVs, and injection scoring, as well as to gather infiltration reporting statistics by physicians.

**Results:**

A total of 1033 injections were monitored at our center. The phase 1 infiltration rate was 2.1%. In decision tree analysis, patients < 132.5lbs were associated with infiltrations. Additional analyses suggested patients > 127.5 lbs. with non-antecubital injections were associated with lower quality injections. Our phase 2 infiltration rate was 1.9%. Comparison of injection score to SUV showed no significant correlation and indicated that only 63% of suspected infiltrations were visible on PET/CT imaging.

**Conclusions:**

Developing a quality improvement plan and monitoring PET injections can lead to reduced infiltration rates. No significant correlation between reference SUVs and injection score provides evidence that determination of infiltration based on PET images alone may be limited. Results also indicate that the number of infiltrated PET injections is under-reported.

## Background

Proper administration of a radiotracer dose is essential to positron emission tomography (PET) image quality and quantification [1–5]. Misadministration or infiltration of the dose results in changes to uptake kinetics which may alter the quantitative assessment of PET data. This can impact cancer patient staging, therapy assessment, treatment planning, and can lead to unnecessary invasive procedures and patient radiation exposure [6–9]. Quality control (QC) efforts ensure accuracy of the administered dose for PET quantification; but no routine QC exists to ensure the administered dose completely enters the patient circulation.

The standard quantitative assessment for fluorodeoxyglucose (18F-FDG) PET imaging is the standard up take value (SUV). This value is calculated from the activity concentration measured by the scanner and normalizing by the patient’s weight and the injected dose (ID). SUV is given by the equation below.

$$ {SUV}_{BW}=\frac{ROI\  Activity\ Concentration}{ID/ Weight} $$

If there are errors in the injected dose value (ID), possibly caused by a compromised injection, then this can introduce significant variance into the calculation of SUV and subsequently can lead to inaccurate assessments of quantitative results that are often used for response to therapy assessments [10].

It is also a common practice for radiologists to report the maximum value of the SUV in the left lobe of the liver as a reference region for a given FDG study. The idea behind this methodology is to provide a baseline value for generic FDG uptake in the body to enable better comparison of baseline values to suspected lesion uptake [11, 12]. These values are also sometimes used to make determinations regarding the quality of the scan based on baseline liver values being too low or too high [13], with an exceptionally low value (an SUV of approximately 1) being anecdotally associated with possible infiltration. This is based on a local survey of radiologists that felt like they had noticed an association of uncharacteristically low SUVs in the liver associated with compromised injections.

This study sought to achieve three primary goals. The first was to use new technology to monitor our injection quality and assess our institutional infiltration rates associated with PET/CT radioisotope injections. The second was to use quality improvement techniques to determine potential contributing factors that could be used to reduce our institutional infiltration rates and implement them to determine their true impact on infiltration rates. The third was to assess whether standard baseline PET reporting methods (e.g., SUV max reported in the liver) are able to differentiate between infiltrated and non-infiltrated scans.

## Patients and methods

This study was carried out in two primary research phases. The first phase was conducted under a quality improvement project for which the University of Tennessee Graduate School of Medicine Institutional Review Board (UTGSM IRB) determined the project did not meet the definition of research as defined by 45 CFR 46.102(d) and classified the initiative as “quality improvement”. In Phase 1 of the quality improvement project, our PET/CT center monitored the injection process of 514 patients with technologists blinded to the injection quality results. Data were analyzed and potential contributing factors were identified using decision tree analysis, with decision trees constructed using 20-fold cross validation with inverse prior weights as the assessment measure (SAS Enterprise Miner, v. 14.1 and v.9.4). A quality improvement plan (QIP) to address these factors was developed and implemented around those targeted factors. In Phase 2 of the QI project, 519 patients were monitored with the technologists unblended and able to immediately see the injection quality results and we re-measured our infiltration rate with adherence to the QIP also assessed. All injections were monitored using an external detector device, called LARA (Lucerno Dynamics, LLC, Cary, North Carolina).

The quality improvement plan focused on two main areas: all patients and patients with lower body weight. For all patients, we implemented the following: (1) use of a blood pressure cuff instead of tourniquets (where possible), (2) contacting patients 24 h prior to their exam to remind them of their appointment and to hydrate well, and (3) questioning patients about water consumption the day of the procedure. For patients less than 135 pounds, technologists applied a warm compress to the injection site for several minutes prior to radiotracer injection.

**Fig. 1:**
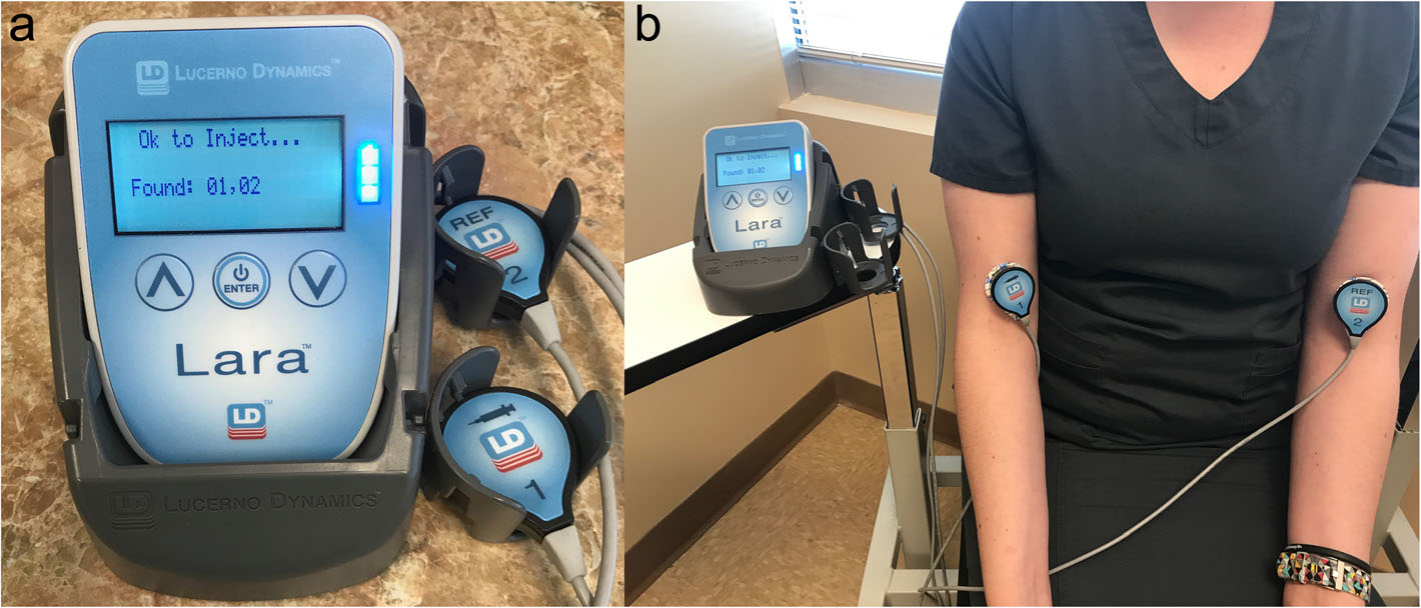
shows (**a**) The Lara device in its docking station, and (**b**) the Lara device and sensors attached to the patient

To monitor the quality of a radiotracer injection, two sensors are placed on the patient using hypoallergenic and atraumatic disposable adhesive pads. One sensor is placed on the injection arm approximately 7 cm proximal to the venous access site. The other sensor is placed on the opposite arm in a mirrored location. Sensors remain in place during the standard resting uptake period prior to imaging (40–60 min post injection). The injection arm sensor records the passage of the bolus and any residual activity at the injection site. The sensor on the opposite arm provides a reference activity level against which the injection sensor is compared. The sensor data, along with procedure-specific information, are analyzed using cloud-based software to generate TACs and QC/QA reports (see Fig. 1 -Lara Device and TAC).

**Fig. 2:**
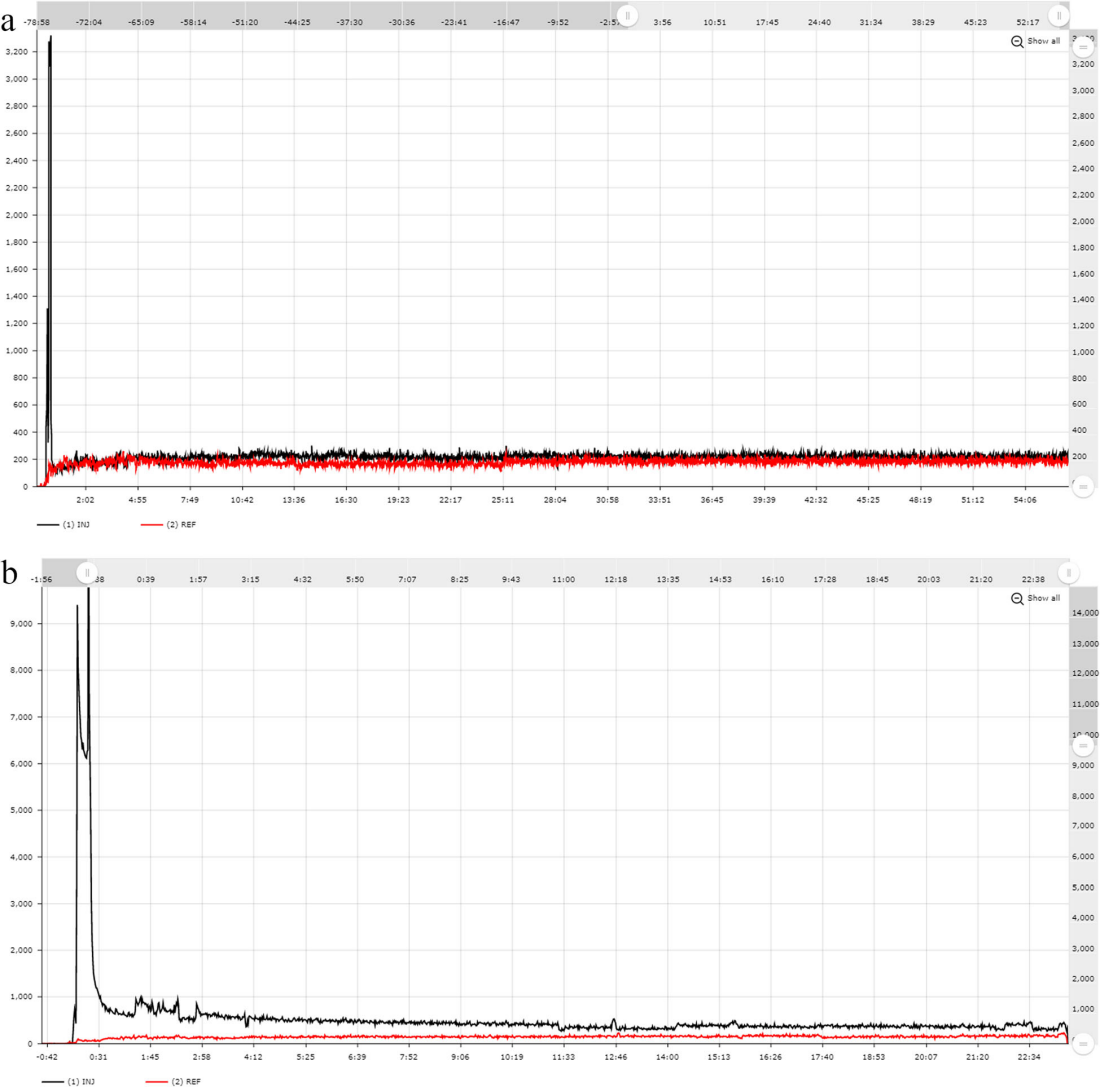
shows a good quality injection (**a**) as compared to an injection of poor quality (**b**)

**Fig. 3:**
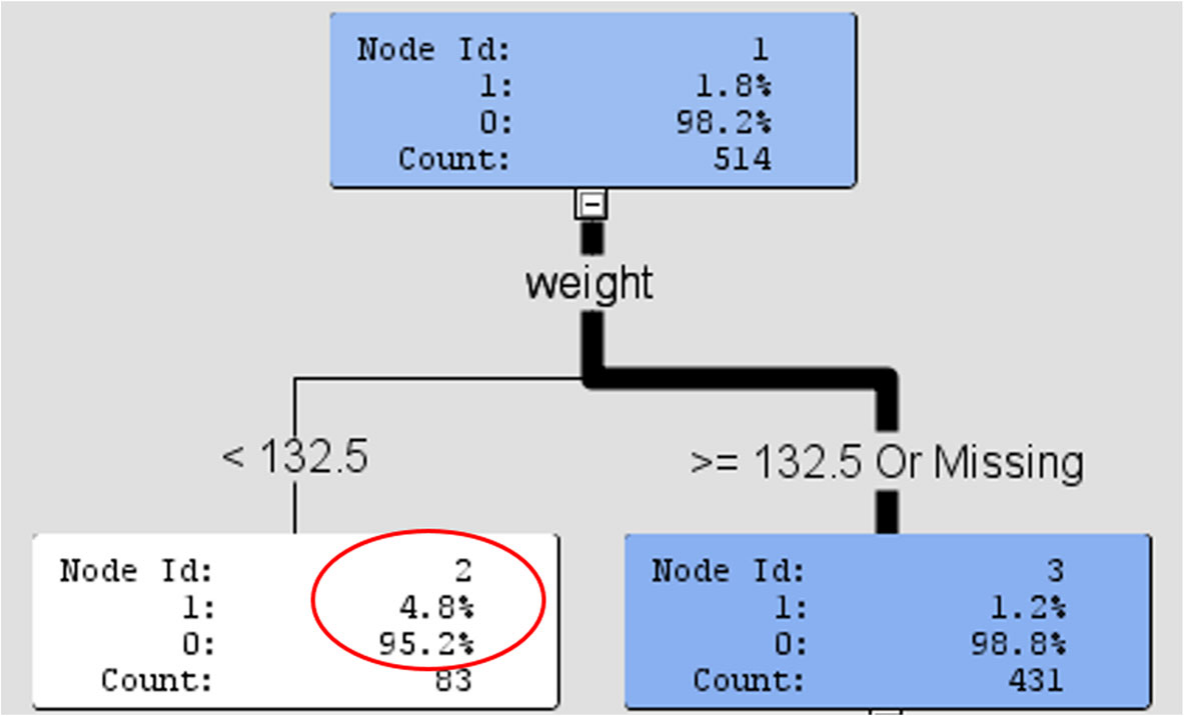
shows the decision tree analysis results for Phase I injection monitoring

For an ideal injection, the TACs reported by the injection sensor should quickly peak and then rapidly approach the values recorded by the reference sensor as shown in Fig. 2a. For injections which may have been compromised by infiltration or a venous obstruction, the activity at the injection site will remain elevated during part or all of the uptake period as shown in Fig. 2b. TACs with the latter characteristics are indicative that not all of the prescribed radioactivity was delivered as a bolus injection into the patient’s circulation. Examples of quality injections and injections with signs of infiltration are shown in Fig. 3.

### SUV analysis and correlation to injection scores

Subsequent to the completion of the QI project, we obtained UTGSM IRB approval (#4365) to retrospectively compare PET/CT imaging data to injection quality results. In this companion study, 896 patients whose injections were monitored had their injection quality scores compared to the radiology reports and images from their PET/CT examination. Values for maximum SUV in the reports were compared to injection quality scores from the device to test for correlations between SUV values and injection scores. Scores of greater than 200 were classified by our site as infiltrations with all remaining scores grouped as good injections. Mann-Whitney U tests were used for comparison of group means and Spearman’s Rho testing was used to assess non-parametric correlation.

In addition to obtaining SUVs from patient reports, we examined the imaging data for cases considered to be potentially infiltrated (score > 200) to determine the percentage of infiltrations that were visible in the PET field of view (FOV) and specifically called out in the radiology reports. For many infiltrations, the site may not be visible in the scanner because of arm positioning, however, we felt this was an important characteristic to determine what percentage of infiltrations could have been missed by our institution had we not externally monitored for injection quality.

## Results

### Infiltration quality improvement project

The infiltration rate at our institution from phase 1 was found to be 2.1% (SE .81, 95% CI 1.02, 4.47). In decision tree analysis (Fig. 2), patients < 132.5lbs were associated with a higher number of suspected infiltrations and were shown to be 4× more likely to be infiltrated (4.85 vs. 1.2%). Additional analyses suggested patients > 127.5 lbs. with non-antecubital injections were associated with lower quality injections. Following implementation of our QI plan, the phase 2 infiltration rate was 1.9% (SE .76, 95% CI .87, 4.16) which was a measurable reduction but not statistically significant (*p* = 0.785). The infiltration rate in patients < 132.5 lbs. decreased from 4.8 to 1.4% (*p* = 0.23) and in patients > 127.5lbs with non-antecubital injections increased from 2.7 to 7.5% (*p* = 0.20) as shown in Table 1. Estimates of compliance with QIP measures ranged from 19 to 45%.

**Table 1:** Associations with Infiltrations and Corresponding Phase 1 and Phase 2 Rates

Associations with Infiltrations	Infiltration Rate Phase 1	Infiltration Rate Phase 2	Change in Rate	*p* Value
Patients < 132.5 lbs	4.8% (4/83)	1.4% (1/72)	71% ↓	0.23
Patients > 127 lbs. with non-antecubital injections	2.7% (2/73)	7.5% (5/67)	177% ↑	0.20

### SUV analysis and correlation to injection scores

Assessment of the correlation between maximum SUVs in the liver and injection scoring indicated a very weak, non-significant correlation between the injection score and SUV with a Spearman’s Rho correlation coefficient of − 0.08 with a *p* value of 0.17. The average liver SUV for patients considered having infiltrated injection was 3.83 with maximum and minimum values of 6.4 and 2.2, respectively. For patients that were not infiltrated, the average liver SUV was 4.04 with maximum and minimum liver values of 12 and 1.7, respectively. A weak but significant correlation was observed between injection score and patient weight (ρ = − 0.125, *p* = 0.040) as well as a weak but significant correlation between blood glucose levels and patient weight (ρ = − 0.168, *p* = 0.006).

Further highlighting the lack of correlation between the injection score and SUVmax values, assessment of the liver SUVmax scores from the twenty worst injections scores and twenty best injection scores indicated that the mean values differed by only 9 % (3.585 ± 0.78 and 3.925 ± 1.12). Two-sample t-tests for means of these two samples were found to not be significant (*p* > 0.05) suggesting that the two means were not significantly different.

Of thirty-eight measured infiltrations during the study period, twenty-four were visible on imaging data while fourteen were not (63% visible on scans). For all scans in which the infiltration was not visible, none were mentioned in the radiology reports. Only in four instances out of twenty-four visible infiltrations were the infiltrations specifically noted in the radiology report. This indicates that during this study, approximately 17% of visible infiltrations were reported, while only 10.5% of the total number of infiltrations were reported by radiologists.

## Discussion

**Fig. 4:**
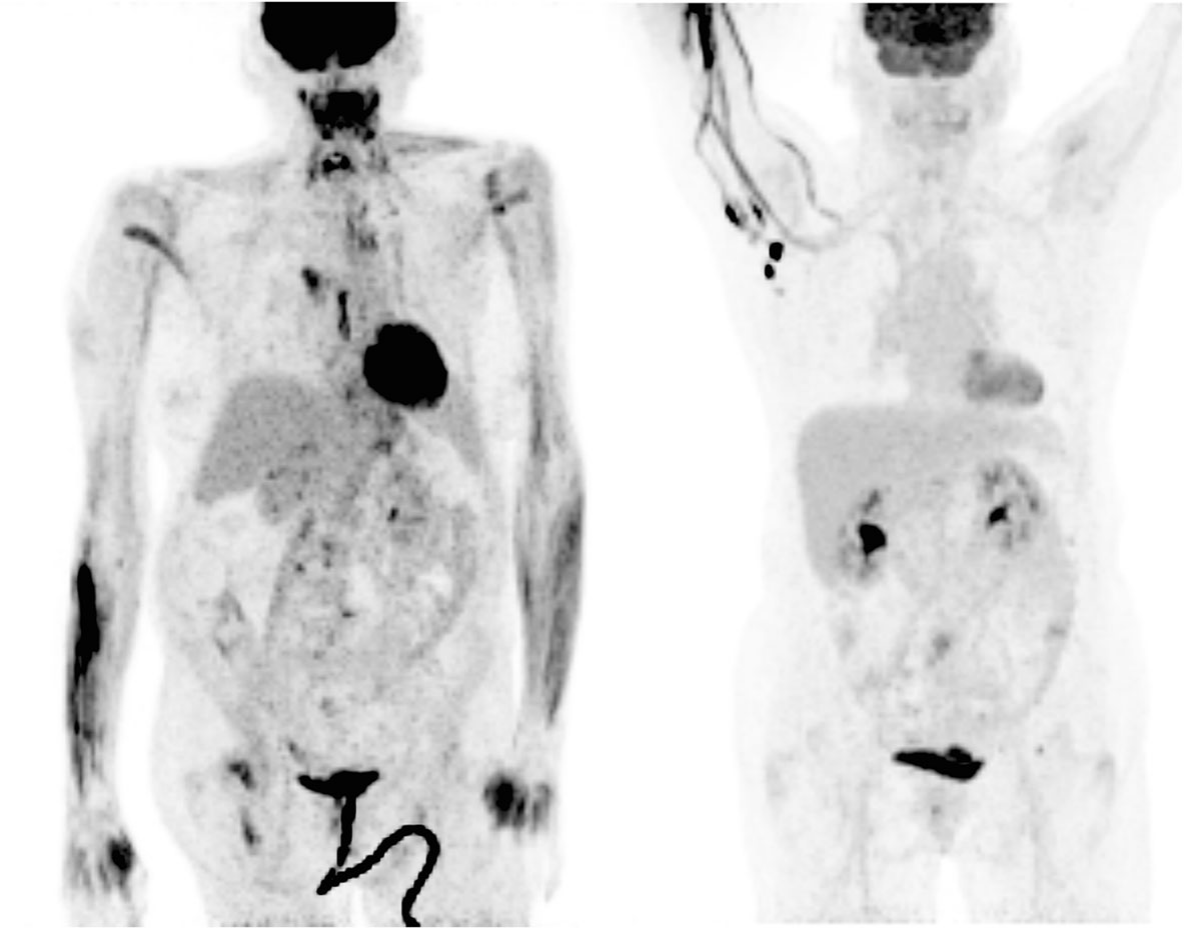
shows two examples of extravasated doses. The left image shows a visible infiltration with abnormal FDG distributions and high image noise related to reduced counts distributed through the patient. The right image shows the infiltration visible in the arm with high nodal uptake that was later determined to only be related to infiltration of the PET tracer dose

No significant correlation was found between SUV maximum measurements in the liver and injection scoring. Contrary to anecdotal and suggested information, there appears to be no predictive correlation between the SUV maximum values assessed in the liver as a reference region and whether or not an infiltration occurred in a PET injection. This is true for the average PET scan, however, the authors concede that severe infiltrations may result in potential visual changes to the data that may make it evident that an issue occurred with the injection. Figure 4 shows two examples of compromised injections. These images show different aspects of altered image quality, including increased image noise, non-normal patterns of 18F-Fluorodeoxyglucose (FDG) uptake, and axillary node involvement combined with image quality issues which is a well-known sign of a possibly infiltrated dose [14].

For diagnostic clinical assessments of PET/CT data, the lack of significant correlation between liver SUV measurements and injection quality results demonstrates that the use of liver SUV information cannot be used as a baseline for assessment of the quality of any individual patient injection. Injection quality monitoring is needed to more positively determine the quality of a given injection so that appropriate assumptions about the integrity of the resulting PET/CT scan can be made. This is especially important in longitudinal therapy monitoring where baseline pre-therapy SUV measurements may have been compromised by poor injection quality and could result in changes to patient management if the compromised SUV comparison to subsequent SUVs factor into the physician determination of appropriate treatment.

Reporting frequency of infiltrations appears to be low. Even in the cases where the infiltration was clearly visible on imaging, only 17% were reported officially on the radiology report. It is our opinion that information about the quality of the injection should be consistently placed into the official radiology report to provide treating physicians with key information regarding potential quality issues related to a metabolic study. Reporting of this information is not a standard practice at many facilities but may improve as access to injection monitoring becomes more readily available and the imaging community becomes more aware of the potential impact unknown infiltrations may have on cancer care.

At our institution, the time activity curve image with the injection score is uploaded to PACS with the PET/CT study images as a secondary capture image. This score is reported on with standardized language, similar to the following text: “The injection quality is good with injection score of -369 (200 or greater suggesting of radiotracer infiltration)”. If the injection score were above 200, we would have the language similar to the following: “The injection may be compromised with and injection score of 300 (200 or greater suggesting of radiotracer infiltration)”. The goal is not to specifically say an injection is absolutely good or bad, but our goal is to alert referring physicians and radiologists to possible compromises to injection quality that is especially useful if quantitative assessments are being used, or longitudinal patient imaging is being performed.

Limitations exist with this study. Firstly, this is a single center experience and is thus biased by our own processes and patient populations and may not reflect outcomes measured by other centers. Secondly, the retrospective portion of this study only enables us to examine the correlations between existing data as no interventions were used to assess further causal relationships. Further work is needed to validate the complete meaning of the data collected using external sensors for the purposes of injection monitoring and quality control. A recent study has validated that results from external sensors match with information recorded during PET imaging [15], however, this study does not identify how the time activity curves from the external sensors match with the kinetics of the infiltration and redistribution into the body. Although this work remains to be performed, the process of adding better quality improvement through injection monitoring undoubtedly can have an impact on patient care in the outpatient cancer imaging setting.

Previous studies, including a recent multi-center center study of 5541 injection (including some data from our site) that indicated injection monitoring can lead to PET center injection quality improvements and can lead to changes in patient management [1, 7, 16]. At our site, poor injection quality occurred at a lower frequency compared to other sites large multi-center study (2.1% for our site, vs. 6.2% average for other sites), however, we were still able to improve upon our injection quality by implementing an appropriate quality improvement plan. We show in this work that even centers with low suspected infiltration rates can benefit from consistent injection monitoring and quality improvement initiatives.

Novel to this work is our detailed assessment of baseline liver values to injection scoring and information on reporting. Other studies have indicated an 11% reduction of infiltrated liver values and hinted that underreporting of compromised is likely present [1]. In this work we found only a weak, non-significant correlation to SUV max liver values with a difference of approximately 5–9% between good and compromised injections, smaller than previously reported. We also quantitatively assessed reporting of infiltrations showing significant underreporting in radiology reports and the need to improve reporting on injection quality to provide the best possible quality of care.

## Conclusions

Previous studies have indicated that infiltration can cause quantitative and visual uncertainty, while this study further illustrates the need for injection quality monitoring by showing that the commonly used reference region of the liver may not be a reliable indicator of the degree of injection infiltration. Injection monitoring, and developing a quality improvement plan can lead to improvements in injection quality for patients. At our center we started with a low infiltration rate of 2.1%, but were able to improve our rates even with those small numbers with a well thought out quality improvement plan based on our specific patient population. For sites with greater infiltration percentages [1], monitoring and development of improvement plans could play a significant role in improving the quality of injections at a given institution.

## Data Availability

All data is available upon request.

## Abbreviations

18F-FDG: 18F-fluorodeoxyglucose
CT: Computed Tomography
ID: Injected Dose
PET: Positron Emission Tomography
QC: Quality Control
QIP: Quality Improvement Plan
ROI: Region of Interest
SUV: Standard Uptake Value
TAC: Time Activity Curve
UTGSM IRB: University of Tennessee Graduate School of Medicine Institutional Review Board
